# EXPLORING GOAL CONTENT AND ORIENTATION OF SWISS CENTENARIANS

**DOI:** 10.1093/geroni/igae098.0219

**Published:** 2024-12-31

**Authors:** Adar Hoffman, Daniela Jopp, Stefano Cavalli, Armin von Gunten, Christina Roecke, Mike Martin, Francois Herrmann

**Affiliations:** University of Lausanne, Lausanne, Vaud, Switzerland; University of Lausanne, Lausanne, Vaud, Switzerland; University of Applied Sciences and Arts of Southern Switzerland, Manno, Ticino, Switzerland; Lausanne University Hospital, Prilly, Vaud, Switzerland; University of Zurich, Zurich, Zurich, Switzerland; University of Zurich, Zurich, Zurich, Switzerland; Geneva University Hospitals, Thônex, Geneve, Switzerland

## Abstract

In very old age, goal orientation is expected to shift towards maintenance-oriented goals, as adaptive mechanism (e.g., Ebner, Freund, & Baltes, 2006). For centenarians, opportunities can be especially limited due to physical and contextual constraints, calling for adjustment in goal content and orientation. This study investigated the number, content, and orientation of goals among a subsample of 135 centenarians (Mage = 101.6 years) in the SWISS100 study. Within this sample, 78 (58%) centenarians mentioned at least one goal, and 57 (42%) centenarians did not mention any. Specifically, 27 (35%) specified one goal, 24 (30%) two, and 27 (35%) three distinct goals. Goals were coded for content and orientation. Leisure and family contents were most prevalent domains for goal setting, covering half of the goals mentioned. Subsequently came contents of health and home-related, while domains of social, well-being, independence and religion were also mentioned but to a lesser extent. Moreover, approach and maintenance orientations were the most widespread, representing 75 percent of all goal orientations. Some improvement goals were mentioned, but avoidance goals were rare, particularly for second and third goals. A higher number of goals was associated with more meaning in life and higher subjective well-being. Overall, results show that centenarians still have goals, the most prevalent contents reflect their life stage, and the many approach goals suggest a positive orientation. As goals seem to remain important mechanism of adaptation among centenarians, more research is needed to better understand the role of goals as a potential means of successful aging.

